# A+-Helix of Protein C Inhibitor (PCI) Is a Cell-penetrating Peptide That Mediates Cell Membrane Permeation of PCI*

**DOI:** 10.1074/jbc.M114.581736

**Published:** 2014-12-08

**Authors:** Hanjiang Yang, Felix Christof Wahlmüller, Bettina Sarg, Margareta Furtmüller, Margarethe Geiger

**Affiliations:** From the ‡ Center of Physiology and Pharmacology, Department of Vascular Biology and Thrombosis Research, Medical University of Vienna, A-1090 Vienna, Austria and; § Biocenter, Division of Clinical Biochemistry, Innsbruck Medical University, A-6020 Innsbruck, Austria

**Keywords:** Cell Permeabilization, Cell-penetrating Peptide (CPP), Nuclear Translocation, Peptides, Serpin, Protein C Inhibitor, Testisin

## Abstract

Protein C inhibitor (PCI) is a serpin with broad protease reactivity. It binds glycosaminoglycans and certain phospholipids that can modulate its inhibitory activity. PCI can penetrate through cellular membranes via binding to phosphatidylethanolamine. The exact mechanism of PCI internalization and the intracellular role of the serpin are not well understood. Here we showed that testisin, a glycosylphosphatidylinositol-anchored serine protease, cleaved human PCI and mouse PCI (mPCI) at their reactive sites as well as at sites close to their N terminus. This cleavage was observed not only with testisin in solution but also with cell membrane-anchored testisin on U937 cells. The cleavage close to the N terminus released peptides rich in basic amino acids. Synthetic peptides corresponding to the released peptides of human PCI (His^1^–Arg^11^) and mPCI (Arg^1^–Ala^18^) functioned as cell-penetrating peptides. Because intact mPCI but not testisin-cleaved mPCI was internalized by Jurkat T cells, a truncated mPCI mimicking testisin-cleaved mPCI was created. The truncated mPCI lacking 18 amino acids at the N terminus was not taken up by Jurkat T cells. Therefore our model suggests that testisin or other proteases could regulate the internalization of PCI by removing its N terminus. This may represent one of the mechanisms regulating the intracellular functions of PCI.

## Teaser

**Background:** Extracellular protein C inhibitor (PCI) can cross the cellular plasma membrane.

**Results:** Testisin (fluid-phase and cell membrane-anchored) cleaves PCI close to its N terminus. N-terminally truncated PCI can no longer be internalized by cells.

**Conclusion:** Testisin removes helix A+, a cell-penetrating peptide, which mediates cell membrane permeation of PCI.

**Significance:** Testisin or other proteases could regulate PCI internalization by removing its N terminus.

## Introduction

Protein C inhibitor (PCI^3^; SerpinA5) is a serine protease inhibitor with broad protease reactivity (1). It belongs to clade A of the serpin (serine protease inhibitor) superfamily (1) and was initially identified in human plasma as an inhibitor of the anticoagulant serine protease activated protein C (aPC) (2). Subsequently, PCI was recognized to be an inhibitor of many other serine proteases including blood coagulation factors (3), fibrinolytic enzymes (4, 5), tissue kallikrein (6), acrosin (7), hepatocyte growth factor activator (8), and enteropeptidase (9). Like other serpins, PCI inhibits its target proteases by forming covalent enzyme-serpin complexes (10). PCI is a heparin-binding serpin, and heparin and other glycosaminoglycans can modulate its activity and target enzyme specificity (10–12). Other non-protease ligands like oxidized/negatively charged phospholipids such as phosphatidylserine, oxidized phosphatidylserine, oxidized phosphatidylethanolamine, and phosphoinositides (13, 14) show heparin-like effects and may enhance the inhibitory activity of PCI (13, 14).

We have shown previously that PCI can be internalized by cells by a phosphatidylethanolamine-dependent mechanism (15). Inside the cell, PCI can translocate to the nucleus. Although we have identified several intracellular proteins interacting with PCI (16, 17), the biological relevance of PCI internalization and nuclear translocation still has to be examined. In addition, glycosaminoglycans and phospholipids present on cell membranes (13, 18) may not only mediate the permeation of PCI through membranes but may also bring PCI into close vicinity of cell membrane-associated serine proteases. To the best of our knowledge, there are only a few publications about the interaction of PCI with membrane-associated serine proteases (9, 19, 20). Among those reports, enteropeptidase is the only membrane-anchored serine protease whose interaction with PCI has been biochemically characterized (9).

The aim of this study was to investigate the interaction of PCI with testisin. Testisin (also referred to as ESP1, TEST5, and PRSS21 (21–23)) is a serine protease that is posttranslationally modified by the addition of a C-terminal glycosylphosphatidylinositol membrane anchor (23, 24). It is highly expressed in male germ cells and sperm (22, 25) and is found in capillary endothelial cells, eosinophils (21), and ovarian cancers (24, 26).

Testisin has been described as a putative non-classical type 2 tumor suppressor in testicular cancer because loss of testisin expression by germ cell-derived testicular tumors has been observed (22). In contrast, testisin is highly expressed in ovarian tumor cells with little or no expression in normal ovaries and is linked to advanced stage disease in primary human ovarian carcinomas (26). Subsequently Tang *et al.* (24) demonstrated that enzymatic activity of testisin is critical for transformation of tumor cells and that knockdown of testisin in ovarian cancer cells leads to increased apoptosis and reduced cell growth in soft agar. Testisin-deficient spermatozoa show abnormal function despite subnormal fertility of testisin-deficient mice (27, 28). Those sperm exhibit decreased mobility, angulated and curled tails, fragile necks, and increased decapitation (28). Those abnormal features may lead to reduced sperm-egg binding and decreased fertility in short term fertility studies (28). Data also show reduced ability of testisin-deficient spermatozoa to bind to the zona pellucida and fuse with eggs *in vitro*. This defect could be rescued by treatment of spermatozoa with uterine fluids (27). Interaction of protease inhibitors with testisin may therefore be not only important in tumor invasion and metastasis but also in the development and maturation of spermatozoa.

We have shown previously that targeted disruption of the PCI gene results in infertility of male homozygous mice that is caused by abnormal spermatogenesis (29). This is not surprising because in adult mice PCI is almost exclusively expressed in the reproductive tract. In contrast, human PCI (hPCI) is expressed in many tissues and is present in blood and most body fluids (30). Very high concentrations of hPCI are present in the male and female reproductive tracts (30), and loss of PCI activity in seminal plasma has been shown to be associated with infertility (31). Recently there has been increasing evidence in the literature that loss of PCI expression in cancer cells is associated with increased malignancy and that PCI could act as a tumor suppressor. There are studies showing that PCI expression is decreased or absent in renal, prostate, and ovarian cancers (32–35). In ovarian tumors, the reduction or loss of PCI expression is associated with a more aggressive phenotype of tumors (33, 34). Two single nucleotide polymorphisms identified in the PCI gene are significantly related with risk of papillary thyroid cancer (36). Growth and metastasis of breast cancer cells are suppressed by incubation with exogenous PCI or by overexpression of PCI independently of its serine protease inhibitory activity (37). PCI inhibits growth and migration of breast cancer cells partially by inhibiting cathepsin L activity (38). The mechanism(s) responsible for the protective effects against tumor growth have not been elucidated so far.

Here we investigated the interaction of PCI with testisin and could show that hPCI and mouse PCI (mPCI) act as substrates for testisin. Both hPCI and mPCI were cleaved by testisin at the reactive center loop and at an additional site close to the N terminus. We furthermore showed that the N-terminal peptides of hPCI and mPCI exhibited cell penetrating activity, mediating the internalization of PCI by Jurkat T lymphoma cells.

## MATERIALS AND METHODS

### 

#### 

##### Cell Culture

Jurkat T lymphoma cells (Clone E6-1, ATCC number TIB-152) and U937 histiocytic lymphoma cells (ATCC number CRL-1593.2) were maintained in RMPI 1640 medium supplemented with 10% fetal bovine serum (Sigma-Aldrich), 20 mm Hepes, 100 μg/ml penicillin, and 100 units/ml streptomycin (full medium) in a humidified atmosphere containing 5% CO_2_ at 37 °C. For the internalization experiment, cells were seeded at 1 × 10^6^ cells/ml with 8 ml of full medium into 75-cm^2^ flasks and treated with respective 200 nm proteins for 2 h or 5 μm peptides for 1 h.

##### Preparation of Recombinant Proteins

Expression vectors of recombinant mouse PCI (rmPCI)^4^ and recombinant human PCI (rhPCI) (39) were obtained from the collection of the Department of Vascular Biology and Thrombosis Research, Medical University of Vienna. Expression vectors of ΔR1-A18 mPCI (lacking the first 18 amino acids corresponding to helix A+), hPCI-R11G (Arg^11^ replaced by Gly), hPCI-R354G (Arg^354^ replaced by Gly), mPCI-A18G (Ala^18^ replaced by Gly), or mPCI-R352G (Arg^352^ replaced by Gly) were constructed as described previously (39). Briefly, mutated mPCI DNA fragments were prepared by PCR (see primers in Table 1) and cloned into the pET-15b plasmid. Constructs were confirmed by sequencing (Microsynth AG, Balgach, Switzerland). Expression and purification of recombinant proteins were performed as described previously (39).

**TABLE 1:** **Primers used in this study** F, forward; R, reverse.

Primer name	Primer sequence
ΔR1-A18 mPCI F	CTGGTGCCGCGCGGCAGCCATATGGTGGGACCTCCCAGTAG
ΔR1-A18 mPCI R	CGGGCTTTGTTAGCAGCCGGATCCTCAGGGCCGGGTCACC
pET15b F	ATGCAAGGAGATGGCGCCC
pET15b R	TATCACGAGGCCCTTTCGTC
hPCI-R11G F	GAAGAAGGGAGTCGAGGACCTCCATGTAGG
hPCI-R11G R	CCTCGACTCCCTTCTTCATCTCCCGGGGG
hPCI-R354G F	TTCGGATCGGCCCGCCTGAACTCTC
hPCI-R354G R	CAGGCGGGCCGATCCGAAAGTGAATATTGTCCCCG
mPCI-A18G F	GAGTCCTCGGTGGGTGGAGTGGGACCTCCCAGTAGC
mPCI-A18G R	CCCACTCCACCCACCGAGGACTCTTTAG
mPCI-R352G F	CACATTCGGATCTGCTCGGCCGAGCTCCC
mPCI-R352G R	CGGCCGAGCAGATCCGAATGTGAAGATGGCTCC

##### Interaction of PCI and PCI Mutants with Testisin

Activation of protestisin and testisin (R&D Systems, Minneapolis, MN) activity assays were performed according to the instructions of the supplier. Inhibition of testisin by PCI was tested on 96-well F16 Black MaxiSorp plates (Nunc, Roskilde, Denmark). 20 nm testisin was incubated with 200 or 1000 nm (final concentration) rhPCI or rmPCI in the absence or presence of 5 units/ml heparin in 100 μl of testisin buffer (50 mm Tris, 150 mm NaCl, 10 mm CaCl_2_, pH 7.5). After a 30-min incubation at 37 °C, 100 μl of 0.2 mm fluorogenic substrate I-1295 (Bachem, Bubendorf, Switzerland) dissolved in testisin buffer was added to each well. Plates were read every 15 s at an excitation wavelength of 380 nm and an emission wavelength of 460 nm using a Synergy H4 microplate reader (BioTek, Seattle, WA). The 5-min time point was used to calculate the relative activity. The amidolytic activity of testisin in the absence of PCI was assigned to 1, and for each reaction, the remaining testisin activity was calculated. To detect the possible complex formation between PCI and testisin, different concentrations (7.5–960 nm) of rhPCI or rmPCI, respectively, were incubated with 30 nm testisin for varying times (0–40 min) at 37 °C. The reactions were stopped by adding an equal volume of 2× Laemmli buffer containing 5% 2-mercaptoethanol (40). Samples were heated at 99 °C for 5 min followed by sodium dodecyl sulfate-polyacrylamide electrophoresis (SDS-PAGE) and Western blotting. To determine the testisin cleavage sites in human and mouse PCI, WT PCI or PCI mutants (rhPCI, hPCI-R11G, hPCI-R354G, rmPCI, mPCI-A18G, or mPCI-R352G, respectively; 300 nm each) were incubated with 30 nm testisin for 1 h at 37 °C. An equal volume of 2× Laemmli buffer containing 5% 2-mercaptoethanol was added to those samples to stop the reaction followed by SDS-PAGE and Western blotting.

##### His Tag Precipitation of Intact rmPCI, Testisin-pretreated rmPCI, or Testisin

**FIGURE 1.**
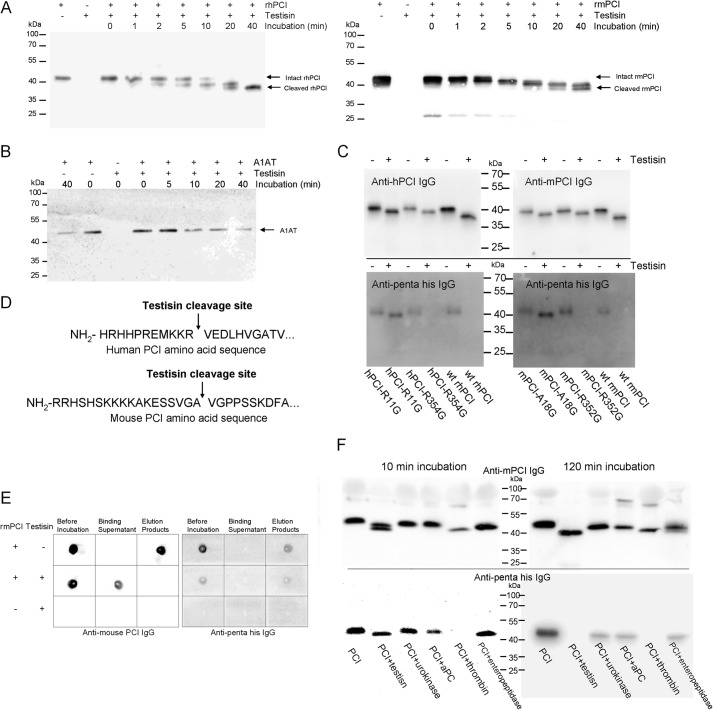
**Cleavage of PCI by testisin.** *A*, rhPCI (*left panel*) or rmPCI (*right panel*; 300 nm each) and testisin (30 nm) were incubated at 37 °C in a volume of 10 μl of testisin buffer for the time periods indicated in the figure. The incubation mixtures were treated with 2× Laemmli buffer containing 5% 2-mercaptoethanol and subjected to SDS-PAGE followed by Western blotting using IgG against hPCI or mPCI, respectively, as described under “Materials and Methods.” *B*, A1AT (300 nm) and testisin (30 nm) were incubated at 37 °C in a volume of 10 μl of testisin buffer for the time periods indicated in the figure. The incubation mixtures were treated with 2× Laemmli buffer containing 5% 2-mercaptoethanol and subjected to SDS-PAGE followed by Western blotting using anti-A1AT IgG as described under “Materials and Methods.” *C*, hPCI-R11G, hPCI-R354G, rhPCI (*left panel*), mPCI-A18G, mPCI-R352G, or rmPCI (*right panel*; 300 nm each) was incubated without or with testisin for 120 min at 37 °C in a volume of 10 μl of testisin buffer. The incubation mixtures were treated with an equal volume 2× Laemmli buffer containing 5% 2-mercaptoethanol and subjected to SDS-PAGE followed by Western blotting using IgG against hPCI, mPCI, or penta-His, respectively, as indicated in the figure. *D*, N-terminal testisin cleavage sites of hPCI and mPCI as determined by Edman degradation. *E*, His tag precipitation of intact rmPCI, testisin-pretreated rmPCI, or testisin. Samples were prepared as described under “Materials and Methods” and spotted on PVDF membrane as indicated in the figure. Membranes were subjected to Western blotting using anti-mPCI IgG (*left panel*) or anti-penta-His IgG (*right panel*), respectively. *F*, mPCI (300 nm) was incubated with testisin, urokinase, aPC, thrombin, or enteropeptidase (30 nm each), respectively, for 10 (*left*) or 120 min (*right*) at 37 °C. Afterward samples were applied to SDS-PAGE followed by Western blotting using anti-mPCI IgG (*upper panel*) or anti-penta-His IgG (*lower panel*).

rmPCI, testisin-pretreated rmPCI, or testisin (Fig. 1*E*, labeled as *Before Incubation*), respectively, was incubated with cobalt beads at 4 °C for 30 min. Thereafter the beads were collected by centrifugation (3 min at 500 × *g* at 4 °C), and the supernatants (Fig. 1*E*, labeled as *Binding Supernatant*) were transferred to separate tubes. Cobalt beads were washed gently three times with PBS. PBS containing 250 mm imidazole was applied to elute proteins or peptides (Fig. 1*E*, labeled as *Elution Products*) from cobalt beads. 2-μl aliquots of each sample (*i.e.* rmPCI, testisin-pretreated rmPCI, and testisin) and from each step (*i.e.* before incubation with cobalt beads, supernatants from the incubation with cobalt beads, and elution with 250 mm imidazole) were directly spotted on polyvinylidene difluoride (PVDF) membrane and subjected to Western blotting using anti-mPCI IgG or anti-penta-His IgG, respectively.

##### Interaction of PCI with Other Proteases

Urokinase, aPC, thrombin, or enteropeptidase (30 nm each) was incubated with 300 nm rmPCI, respectively, for different periods (10 or 120 min) at 37 °C. The reactions were stopped by adding an equal volume of 2× Laemmli buffer containing 5% 2-mercaptoethanol (40). Samples were heated at 99 °C for 5 min followed by SDS-PAGE and Western blotting.

##### Analysis of α_1_-Antitrypsin (A1AT) Interaction with Testisin

To analyze whether testisin also interacted with other serpins, 30 nm testisin was incubated with 300 nm serum-derived human A1AT (Sigma-Aldrich) for different time periods (0–40 min) at 37 °C. The reactions were stopped by adding an equal volume of 2× Laemmli buffer containing 5% 2-mercaptoethanol (40). Samples were heated at 99 °C for 5 min followed by SDS-PAGE and Western blotting.

##### Inhibition of aPC by PCI

Inhibition of aPC amidolytic activity by PCI was tested on 96-well microtiter plates. 0.2 nm aPC was incubated with 10 or 50 nm (final concentration) rhPCI, rmPCI, hPCI-R11G, hPCI-R354G, mPCI-A18G, mPCI-R352G, or ΔR1-A18 mPCI, respectively, in the absence or presence of 5 units/ml heparin in 100 μl of aPC buffer (25 mm Hepes, 137 mm NaCl, 3.5 mm KCl, 3 mm CaCl_2_, 1% BSA, pH 7.4). After a 30-min incubation at 37 °C, 100 μl of 0.4 mm chromogenic substrate S-2366 (Chromogenix, Milano, Italy) dissolved in aPC buffer was added to each well. The absorbance at 405 nm was determined in a Synergy H4 microplate reader. The 30-min time point was used to calculate the relative activity. The amidolytic activity of aPC in the absence of PCI was assigned to 1, and for each reaction, the remaining aPC activity was calculated.

##### Interaction of PCI with Cellular Testisin

U937 cells (1 × 10^7^ cells/ml) were incubated in RMPI 1640 medium without or with 2 units/ml phosphoinositide phospholipase C (PI-PLC; Calbiochem) at 37 °C for 30 min. Media and cells were separated by centrifugation (5 min at 500 × *g* at 4 °C). For Western blotting experiments, the cells were washed and resuspended in CelLytic cell lysis buffer (Sigma-Aldrich), and the media were concentrated (20–25 times) with Millipore Amicon Ultra-4 centrifugal filter units with Ultracel-10 membrane. Samples were boiled with an equal amount of 2× Laemmli buffer containing 2-mercaptoethanol followed by SDS-PAGE and Western blotting using anti-testisin IgG. For rmPCI cleavage experiments, media were collected, and cells were resuspended in RMPI 1640 medium at a final concentration of 1 × 10^7^ cells/ml. 20 nm rmPCI was incubated with those media or cell suspensions at 37 °C for 0–4 h. After incubation, samples were mixed with 2× Laemmli buffer containing 2-mercaptoethanol and heated at 99 °C for 5 min followed by SDS-PAGE and Western blotting using anti-mPCI IgG.

##### SDS-PAGE and Western Blotting

SDS-PAGE was performed according to the method of Laemmli (40) using 10% acrylamide gels. Following electrophoresis, the proteins were transferred onto PVDF membranes. The membranes were blocked with blotting buffer (5% skim milk powder and 0.05% Tween 20 in PBS, pH 7.4) for 1 h at room temperature. Rabbit anti-mPCI IgG (diluted 1:1000; homemade), rabbit anti-hPCI IgG (diluted 1:1000; homemade), monoclonal mouse anti-penta-His IgG (diluted 1:500; Qiagen, Hilden, Germany), rabbit anti-testisin IgG (diluted 1:500; Abgent, San Diego, CA), mouse monoclonal anti-A1AT(diluted 1:2000; Fisher Scientific GmbH), rabbit anti-actin IgG (diluted 1:2000; Sigma-Aldrich), monoclonal mouse anti-lamin B2 IgG (diluted 1:2000; Abcam, Cambridge, UK), and monoclonal mouse anti-Hsp 90 IgG (diluted 1:2000; Exbio, Prague, Czech) were used, respectively, in blotting buffer. The respective secondary antibodies were horseradish peroxidase-conjugated donkey anti-rabbit IgG (diluted 1:5000; GE Healthcare Handels GmbH) and horseradish peroxidase-conjugated sheep anti-mouse IgG (diluted 1:5000; GE Healthcare Handels GmbH). The signal was detected using SuperSignal West Femto or SuperSignal West Pico chemiluminescent substrate (Thermo Fisher Scientific, Rockford, IL) with a FluorChem HD2 imaging system (ProteinSimple, Santa Clara, CA).

##### Cell Lysis and Subcellular Fractionation

Jurkat T cells were incubated in full medium with 200 nm rmPCI, testisin-cleaved rmPCI, or ΔR1-A18 mPCI, respectively, at 37 °C for 2 h. 1 × 10^7^ cells were harvested and washed three times with PBS. To prepare whole cell lysates, cell pellets were resuspended and incubated in CelLytic M cell lysis buffer at room temperature for 10 min and applied to electrophoresis. Subcellular fractions were prepared as described previously (41) with some modifications. In brief, cell pellets were resuspended in 125 μl of buffer A (10 mm Hepes, pH 7.9, 10 mm KCl, 1.5 mm MgCl_2_, 0.34 m sucrose, 10% glycerol, 1 mm DTT, 0.1% Triton X-100) containing 10 μl of protease inhibitor mixture P8340 (Sigma-Aldrich) and incubated for 8 min. Intact nuclei were collected by low speed centrifugation (5 min at 1300 × *g* at 4 °C), and the supernatant (cytosol) was transferred to another tube. The nuclei were washed once with buffer A plus 110 volume P8340 and then lysed in 125 μl of hypotonic buffer B (3 mm EDTA, 0.2 mm EGTA, 1 mm DTT, 110 volume P8340) for 30 min. Insoluble chromatin and the soluble fraction (soluble nucleus) were separated by centrifugation (5 min at 1700 × *g* at 4 °C). Insoluble chromatin, which was washed once with buffer B, was suspended in 125 μl of micrococcal nuclease buffer (10 mm Tris, pH 9.5, 10 mm KCl, 1 mm CaCl_2_) containing 5 units of micrococcal nuclease (Sigma-Aldrich) and incubated at 37 °C for 15 min. 12.5 μl of 10 mm EGTA (1 mm final concentration) was added to the mixture to stop the reaction. Nuclease-digested chromatin (chromatin-bound proteins) and nuclease-resistant (nuclear envelope) components were separated by centrifugation (5 min at 1700 × *g* at 4 °C). The nuclease-resistant, insoluble fraction (nuclear envelope) was solubilized in 125 μl of 1× Laemmli buffer. If not mentioned, all fractionation procedures were performed on ice.

##### Analysis of the Internalization of Fluorescein Isothiocyanate (FITC)-labeled Peptides by Jurkat T Cells

Human A+-helix peptide (HRHHPREMKKR; corresponding to the cleaved N-terminal peptide of hPCI), mouse A+-helix peptide (RRHSHSKKKKAKESSVGA; corresponding to the cleaved N-terminal peptide of mPCI), and TAT peptide (GRKKRRQRRRPQ) (42) were synthesized and labeled with FITC (GenScript, Piscataway, NJ). Jurkat T cells were incubated with 5 μm human A+-helix-FITC, mouse A+-helix-FITC, TAT-FITC, dextran-FITC, or free FITC, respectively, at 37 °C for 1 h, and cells were washed and fixed with 4% paraformaldehyde for 20 min at room temperature. Fluorescence-activated cell sorting (FACS) analysis was performed with a FACSCalibur flow cytometer (BD Biosciences). Further data analysis was done with Flowing Software (43). For confocal microscopy, the fixed cells were suspended with Vectashield mounting medium containing 1.5 ng/ml DAPI (Vector Laboratories, Burlingame, CA) and transferred onto slides. Confocal images were taken by a Zeiss LSM-510 Meta confocal microscope system (Zeiss, Jena, Germany). Zeiss LSM Image Browser software was applied for further analysis of images.

##### Edman Degradation

Peptide sequencing was performed on an Applied Biosystems Inc. Model 492 Procise protein sequenator. The proteins were blotted onto PVDF membranes, and typically samples were run for 5–10 cycles as required for an unambiguous identification.

## RESULTS

### 

#### 

##### PCI Is a Testisin Substrate

We analyzed the interaction between PCI and testisin by SDS-PAGE followed by Western blotting using anti-PCI IgGs. Incubation of PCI with testisin resulted in a time-dependent decrease in the molecular weight of PCI. No complex formation of PCI with testisin was observed (Fig. 1*A*). To confirm the hypothesis that there is no complex formation between PCI and testisin, we also used antibodies against testisin, but none of the available antibodies was suitable for mouse testisin. Additionally we evaluated the possible inhibition of testisin by PCI using a fluorogenic substrate. PCI was not able to inhibit testisin either in the absence or in the presence of heparin (not shown). To investigate whether testisin interacts with other serpins, we incubated testisin with different concentrations of A1AT for different time periods at 37 °C. Neither cleavage of A1AT nor testisin-A1AT complex formation was detected by Western blotting (Fig. 1*B*).

##### Testisin Cleavage of PCI Leads to N-terminal Peptide Release

As shown in Fig. 1*A*, testisin-cleaved PCI is about 4–5 kDa smaller than intact PCI, suggesting that the potential cleavage site(s) are located close to the C terminus and/or N terminus of PCI. To determine the testisin cleavage site within the PCI molecule, we performed Western blotting using anti-penta-His IgG, which recognizes the N-terminal His tag of recombinant PCI, and aPC inhibition assay to determine whether testisin-cleaved PCI contains a functional reactive site. Anti-penta-His IgG recognized intact PCI but not testisin-cleaved PCI (Fig. 1*C*). Testisin-cleaved PCI did not inhibit aPC either in the absence or in the presence of heparin (not shown). These data suggest that testisin cleaves PCI at two sites: at the N terminus and at the reactive site close to the C terminus. To confirm a cleavage of PCI by testisin close to its N terminus, Edman degradation was performed. New N-terminal sequences of hPCI and mPCI were identified, demonstrating that testisin cleaved hPCI between Arg^11^ and Val^12^ and mPCI between Ala^18^ and Val^19^, respectively (Fig. 1*D*). To confirm and validate these results, we performed mutagenesis of both the N-terminal and the C-terminal cleavage sites in human and mouse PCI and studied cleavage of these mutants by testisin. As can be seen from Fig. 1*C*, testisin-treated human and mouse PCIs in which either the N-terminal or the C-terminal cleavage sites were mutated exhibited a higher molecular weight as compared with the cleaved wild type forms, suggesting that the introduced mutations prevented cleavage at the respective sites. Furthermore testisin-treated N-terminally mutated human (hPCI-R11G) and mouse (mPCI-A18G) PCIs were still recognized by anti-penta-His IgG, which was not the case with wild type and C-terminally mutated human and mouse PCI (Fig. 1*C*, *lower panels*). The peptides that are generated by testisin cleavage of PCI correspond essentially to the A+-helix of hPCI and mPCI, respectively. His tag precipitation revealed that with intact PCI both PCI antigen and the His tag were present in the fraction bound to and eluted from the cobalt beads, whereas with testisin-treated PCI, only the His tag was detected in this fraction, and PCI antigen was found in the “binding supernatant” (=unbound fraction). These data suggest that the A+-helix peptide can be separated from the mPCI core domain after testisin cleavage (Fig. 1*E*). To study whether the N terminus of PCI could be released by other proteases, we also incubated PCI with urokinase, aPC, thrombin, or enteropeptidase for 10 or 120 min, respectively. As shown in (Fig. 1*F*), incubation of mPCI with urokinase, aPC, or enteropeptidase, respectively, also resulted in the appearance of a cleaved PCI form. However, the bands seen after 2-h incubation had a slightly higher molecular weight as compared with the band seen with testisin treatment. Furthermore they were still recognized by anti-penta-His IgG. These data suggest that urokinase, aPC, and enteropeptidase do not cleave the N terminus of mPCI, although we cannot completely rule out the presence of an N-terminally cleaved mPCI form in the aPC-PCI complex because of the lower sensitivity of the anti-penta-His antibody as compared with the anti-mPCI IgG. Here thrombin was used as a control because there is a thrombin cleavage site at the N terminus of the recombinant protein from pET-15b vector.

##### PCI Is Cleaved by Testisin on U937 Cells

**FIGURE 2.**
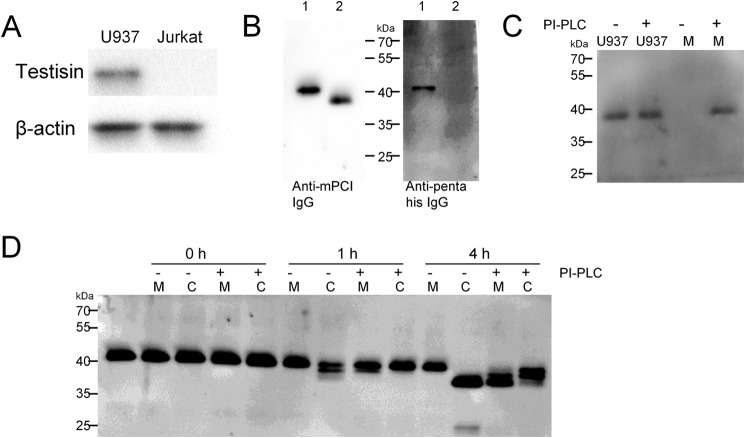
**Cleavage of PCI by U937 cells.** *A*, SDS-PAGE and Western blotting of U937 cell lysate and Jurkat T cell lysate using anti-testisin IgG. *B*, 20 nm rmPCI was incubated in RMPI 1640 medium without (*lanes 1*) or with (*lanes 2*) U937 cells (1 × 10^7^ cells/ml) for 4 h. The conditioned media were separated from cells by centrifugation (5 min at 500 × *g* at 4 °C) and boiled with an equal amount of 2× Laemmli buffer containing 5% 2-mercaptoethanol. The samples were subjected to SDS-PAGE followed by Western blotting using anti-mPCI IgG or anti-penta-His IgG. *C* and *D*, U937 cells were pretreated with PI-PLC for 30 min, and conditioned media were prepared as described under “Materials and Methods.” *C*, U937 cells treated without or with PI-PLC and concentrated media (*M*) were applied to SDS-PAGE followed by Western blotting using anti-testisin IgG. *D*, 20 nm rmPCI was incubated with U937 cells (*C*; untreated or pretreated with PI-PLC) or media (*M*) from these cells at 37 °C for the time periods indicated in the figure. Thereafter samples were subjected to SDS-PAGE and Western blotting using anti-mPCI IgG.

The experiments above have shown that PCI is cleaved by testisin in solution in purified systems. Because testisin is a glycosylphosphatidylinositol-anchored protein, we also analyzed whether this cleavage can occur on the cell membrane. We incubated U937 cells, which express testisin (Fig. 2*A*), with rmPCI. As shown in Fig. 2*B*, rmPCI incubated with U937 cells is cleaved (*left panel*) and cannot be detected with anti-penta-His IgG (*right panel*), suggesting that the N terminus of rmPCI was cleaved by incubation with U937 cells. We used PI-PLC to release testisin from the membrane. As can be seen from Fig. 2*C*, testisin can be detected in the conditioned medium of PI-PLC-treated U937 cells. PI-PLC treatment largely reduced the cleavage of rmPCI by U937 cells (Fig. 2*D*): PI-PLC-pretreated U937 cells or media from untreated cells did not affect the molecular weight of mPCI upon incubation for 1 and 4 h as compared with control. Incubation of mPCI with media from PI-PLC-pretreated U937 cells led to partial degradation of PCI after 1 and 4 h, indicating that cells preincubated with PI-PLC lost membrane-anchored testisin. After 4, h the PCI cleaving activity on the cell surface seemed to be partially recovered.

##### The A+-helix of PCI Is Necessary for the Internalization of the Serpin by Jurkat T Cells

**FIGURE 3.**
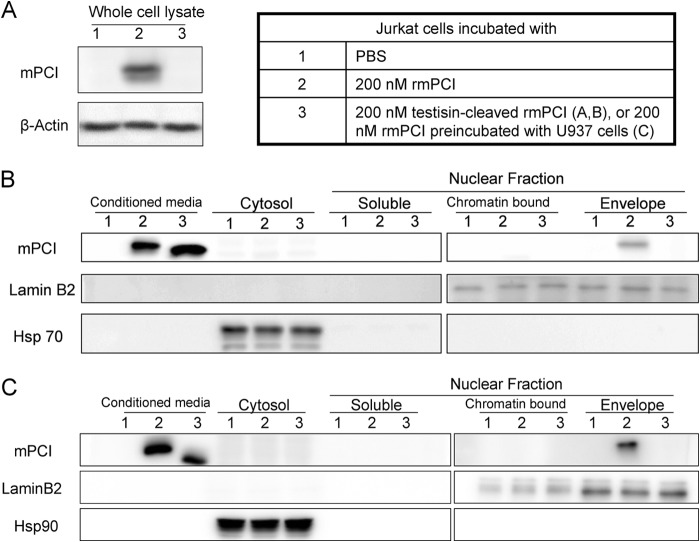
**Internalization of intact mPCI, testisin-cleaved mPCI, and mPCI preincubated with U937 cells by Jurkat T cells.** Jurkat T cells were incubated with PBS (*lanes 1*), 200 nm (final concentration) mPCI (*lanes 2*), testisin-pretreated mPCI (*A* and *B*, *lanes 3*), or mPCI preincubated with U937 cells (*C*, *lanes 3*) for 2 h. Cell lysates and subcellular fractions were prepared as described under “Materials and Methods.” The whole cell lysates (*A*) and subcellular/nuclear fractions (*B* and *C*) were analyzed by SDS-PAGE and Western blotting. IgGs against mPCI, β-actin, lamin B2, or Hsp 90, respectively, as indicated in the figure were applied.

Previous studies have shown that the absence of the A+-helix does not influence the inhibitory activity or the heparin binding of PCI (44, 45). The released A+-helix peptides are rich in basic amino acids, one of the characteristics for cell-penetrating peptides (42). In a previous study, we have shown that PCI is internalized by cells in a phosphatidylethanolamine-dependent manner and can translocate to the nucleus (15). Therefore we hypothesized that the A+-helix peptides might play a role in cell membrane translocation of PCI. To verify this, we first analyzed the A+-helix peptides of hPCI and mPCI using an on line cell-penetrating peptide analysis tool (46). The A+-helix peptides of hPCI and mPCI scored 0.71 and 1.44, respectively, which indicate a high probability of cell-penetrating peptide function. We therefore investigated whether testisin-cleaved PCI or PCI preincubated with testisin-expressing U937 cells could still be internalized by cells. In all internalization experiments, we used mPCI and human cells (Jurkat T cells) to be able to differentiate between internalized PCI and endogenous PCI. As a positive control, we used intact rmPCI, which was taken up by Jurkat T cells and translocated to the nucleus (Fig. 3). In contrast, testisin-cleaved rmPCI could not be detected in the whole cell lysate (Fig. 3*A*) or in any subcellular fraction (Fig. 3*B*). Also rmPCI preincubated with U937 cells was not detected inside Jurkat T cells (Fig. 3*C*).

**FIGURE 4.**
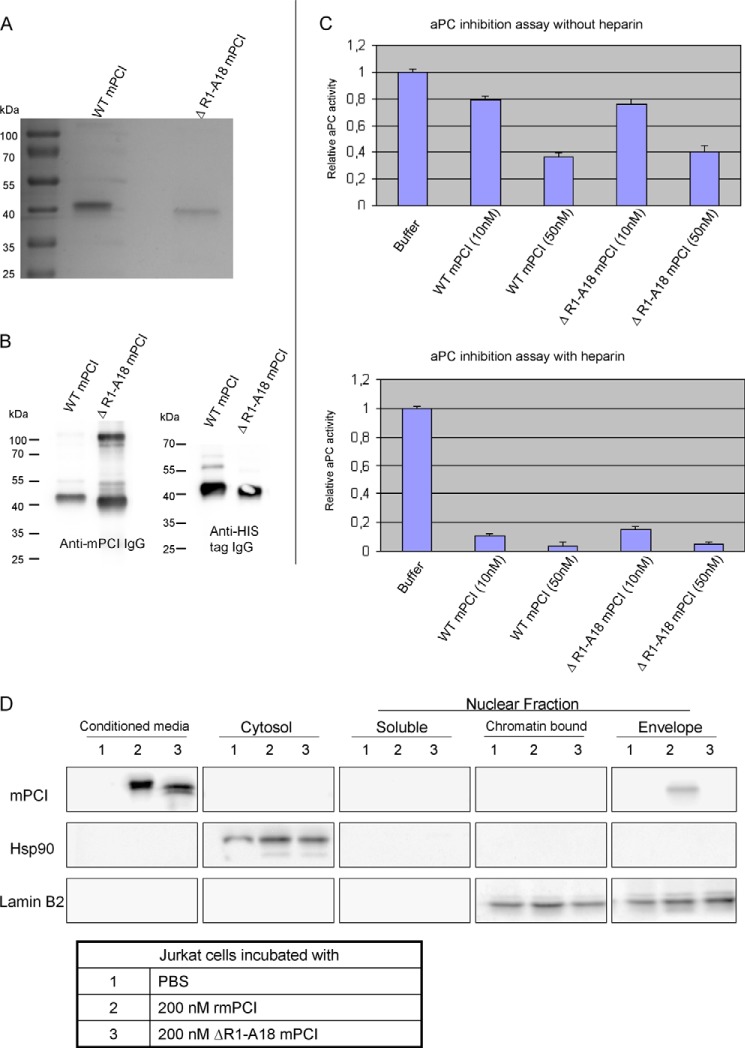
**Purification and characterization of truncated mPCI lacking the N terminus and its internalization by Jurkat T cells.** *A*, Coomassie Blue R-250-stained SDS-polyacrylamide gels of purified WT mPCI (45.8 kDa) and ΔR1-A18 mPCI (43.8 kDa). *B*, WT mPCI and ΔR1-A18 mPCI were analyzed by SDS-PAGE and Western blotting with rabbit anti-mPCI IgG (*left panel*) and anti-penta-His IgG (*right panel*). *C*, inhibitory activity of WT mPCI and ΔR1-A18 mPCI. aPC (1 nm) was incubated for 30 min at 37 °C without or with different concentrations (10 and 50 nm) of WT mPCI or ΔR1-A18 mPCI in the absence (*upper panel*) or presence of heparin (5 units/ml; *lower panel*), respectively. The activity of aPC was determined from the cleavage of S-2366. Data shown in the *graph* are means from three independent experiments, and *error bars* represent S.E. *D*, Jurkat T cells were incubated with PBS (*lanes 1*), 200 nm mPCI (*lanes 2*), or 200 nm ΔR1-A18 mPCI (*lanes 3*), respectively, for 2 h at 37 °C. Cell lysates and subcellular fractions were prepared and analyzed by SDS-PAGE and Western blotting. IgGs against mPCI, lamin B2, or Hsp 90, respectively, as indicated in the figure were applied.

From these experiments, we cannot exclude that cleavage of mPCI at other than the Ala^18^-Val^19^ site (*e.g.* at the reactive site) and/or another reaction of testisin interferes with PCI internalization. We therefore expressed and purified truncated mPCI (ΔR1-A18 mPCI; lacking the N-terminal A+-helix) to mimic testisin-cleaved mPCI (Fig. 4*A*). The recombinant protein contained a His tag at the N terminus. As shown in Fig. 4*B*, the mutant was recognized by anti-mPCI IgG as well as by anti-penta-His IgG. It was functionally active toward aPC, and its activity was stimulated by heparin (Fig. 4*C*). Subcellular fractions were prepared from Jurkat T cells incubated with rmPCI or ΔR1-A18 mPCI and applied to Western blotting using anti-mPCI IgG (Fig. 4*D*). rmPCI was detected in the nuclear envelope fraction of Jurkat T cells (Fig. 4*D*). ΔR1-A18 mPCI was not detected in any of the subcellular fractions (Fig. 4*D*), thereby verifying that the A+-helix peptide of mPCI is essential for its internalization by Jurkat T cells.

##### The A+-helix of PCI Is a Cell-penetrating Peptide

**FIGURE 5.**
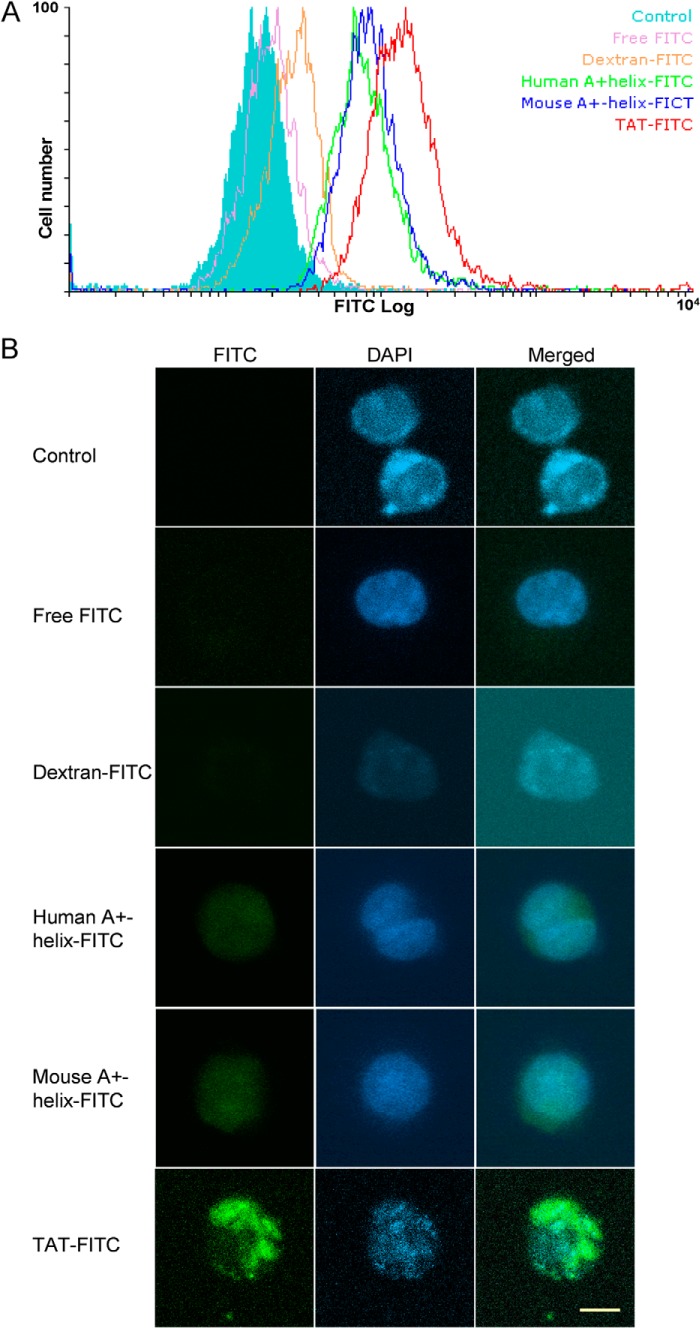
**Internalization of A+-helix-FITC by Jurkat T cells.** Jurkat T cells were incubated for 1 h with 5 μm human A+-helix-FITC peptide, mouse A+-helix-FITC peptide, TAT-FITC peptide, dextran-FITC, or free FITC, respectively. After washing and fixation by paraformaldehyde, cellular fluorescence was analyzed by FACS (*A*) or confocal microscopy (*B*). Cell nuclei were stained with DAPI (*blue*). The *scale bar* represents 5 μm (40×; numerical aperture, 1.3; oil objective).

Although PCI lacking the A+-helix cannot be internalized by cells, we could not conclude that the A+-helix peptide of PCI has cell-penetrating properties. To verify this, we studied the internalization of synthesized FITC-labeled human and mouse A+-helix peptides by cells. FITC-labeled HIV TAT peptide, a well known cell-penetrating peptide (42); free FITC; and dextran-FITC were used as controls. Jurkat T cells treated with FITC-labeled human and mouse A+-helix peptides or FITC-labeled TAT peptides were analyzed by FACS and confocal microscopy and compared with untreated cells. As a negative control, cells treated with an equal molar amount of free FITC or dextran-FITC, respectively, were used (Fig. 5). FACS analysis revealed strong fluorescence signals in cells treated with human or mouse A+-helix-FITC, and an even stronger signal in cells incubated with TAT-FITC was observed. Cells treated with free FITC or dextran-FITC showed a small shift compared with untreated cells (Fig. 5*A*). Confocal microscopy analysis of Jurkat T cells treated with human or mouse A+-helix peptides, respectively, revealed that the A+-helix peptides exhibited cytosolic and nuclear localization (Fig. 5*B*).

## DISCUSSION

In the present study, we investigated the interaction of PCI with testisin. We could show that mPCI and hPCI are cleaved by testisin but do not form SDS-stable complexes with the protease. Consistently we were not able to detect inhibition of testisin by PCI. Testisin-cleaved PCI had no inhibitory activity toward aPC, suggesting cleavage of PCI at its reactive site. In addition, testisin also cleaved PCI close to its N terminus. As judged from Edman degradation of the remaining core protein, this cleavage occurred between Ala^18^ and Val^19^ in mPCI and between Arg^11^ and Val^12^ in hPCI, resulting in the release of a peptide essentially corresponding to the A+-helix of PCI. The fact that testisin cannot remove the N terminus of mPCI-A18G and hPCI-R11G further confirms those new cleavage sites (Fig. 1*D*). We could show cleavage of PCI not only for testisin in solution but also for glycosylphosphatidylinositol-anchored testisin on U937 cells, suggesting that it may also occur *in vivo*. Other proteases that cleave PCI at the reactive center loop site do not trim the N terminus of PCI (Fig. 1*F*). This suggests that if the A+-helix has a certain function this function would be regulated by testisin. The A+-helix is rich in basic amino acids and has been suggested to play a role in heparin binding (47). However, later studies using rhPCI mutants have shown that the H-helix but not the A+-helix functions as the heparin binding site of PCI (44). The A+-helix is not necessary for the inhibitory activity of PCI because hPCI lacking the A+-helix sequence has inhibitory activity similar to that of wild type hPCI (45). Also mPCI lacking A+-helix has inhibitory activity similar to that of intact PCI and can be stimulated by heparin (Fig. 4*C*).

We have shown before that PCI can penetrate through cell membranes in a process requiring the membrane phospholipid phosphatidylethanolamine (15). PCI contains a functional nuclear localization signal,^5^ and once inside the cell, PCI translocates to the nucleus (15). At present, it is still too early to speculate about intracellular functions of PCI; however, we have identified several intracellular/nuclear proteins that interact with PCI (16, 17).

In the human system, PCI is present in many tissues and in most body fluids. Assuming that PCI has in fact intracellular functions, its permeation through the phospholipid bilayer has to be regulated by a mechanism(s) allowing cell membrane penetration of PCI into certain cells while excluding it from others. Peptides rich in basic amino acids such as the A+-helix of PCI have been shown to function as cell-penetrating peptides because of their ability to cross phospholipid membranes. We therefore hypothesized that the A+-helix might be involved in PCI internalization and that testisin, a protease that removes the A+-helix of PCI, could prevent PCI internalization. Because we have identified intracellular interaction partners of PCI in Jurkat T cells (16, 17), we used these cells to study internalization of PCI and of different truncated PCI forms. We can show that PCI pretreated with testisin or preincubated with testisin-expressing U937 cells is no longer internalized by Jurkat T cells. Also a truncated mPCI mutant lacking the A+-helix is not internalized by Jurkat T cells. To further support the hypothesis that the A+-helix of PCI is responsible for PCI internalization, we used FITC-labeled synthetic peptides with the amino acid sequences of the A+-helix of hPCI and mPCI, respectively, and studied their internalization into Jurkat T cells. We could show by flow cytometry as well as by confocal laser-scanning microscopy that these peptides are taken up by Jurkat T cells.

Taken together our results indicate that the A+-helix of PCI is a cell-penetrating peptide that mediates the internalization of PCI by cells. Our data furthermore suggest that proteases such as testisin could regulate the process of PCI internalization through removal of the A+-helix of PCI. This mechanism could be relevant in the male reproductive tract because PCI and testisin are both expressed in the testis, and their absence results in male infertility (29) or subfertility (27, 28), respectively. Furthermore it might play a role in cancer cells where PCI expression is generally associated with less malignant behavior, whereas testisin expression is often higher in malignant cells as compared with their normal counterparts. Further studies are needed to determine the biological role of intracellular PCI and hence the relevance of regulating its internalization by testisin.

## Footnotes

4 M. Furtmüller, unpublished data.

5 B. Sokolikova, J. M. Malleier, F. C. Wahlmüller, J. Breuss, D. Rieger, K. Einfinger, M. Furtmüller, and M. Geiger, manuscript in preparation.

## Footnotes

3 The abbreviations used are:

PCI: protein C inhibitor
mPCI: mouse PCI
hPCI: human PCI
rmPCI: recombinant mPCI
rhPCI: recombinant hPCI
PI-PLC: phosphoinositide phospholipase C
aPC: activated protein C
A1AT: α_1_-antitrypsin
TAT: *transactivator of transcription*.
